# Hub-driven remote synchronization in brain networks

**DOI:** 10.1038/s41598-017-09887-7

**Published:** 2017-09-04

**Authors:** Vladimir Vlasov, Angelo Bifone

**Affiliations:** Center for Neuroscience and Cognitive Systems, Istituto Italiano di Tecnologia, Corso Bettini, 31, I-38068 Rovereto, Italy

## Abstract

The phenomenon of “remote synchronization” (RS), first observed in a star network of oscillators, involves synchronization of unconnected peripheral nodes through a hub that maintains independent dynamics. In the RS regime the central hub was thought to serve as a passive gate for information transfer between nodes. Here, we investigate the physical origin of this phenomenon. Surprisingly, we find that a hub node can drive remote synchronization of peripheral oscillators even in the presence of a repulsive mean field, thus actively governing network dynamics while remaining asynchronous. We study this novel phenomenon in complex networks endowed with multiple hub-nodes, a ubiquitous feature of many real-world systems, including brain connectivity networks. We show that a change in the natural frequency of a single hub can alone reshape synchronization patterns across the entire network, and switch from direct to remote synchronization, or to hub-driven desynchronization. Hub-driven RS may provide a mechanism to account for the role of structural hubs in the organization of brain functional connectivity networks.

## Introduction

Synchronization of oscillatory units is a pervasive phenomenon that is responsible for the emergence of collective behaviors in natural and artificial systems^1^. Entrainment of these dynamical systems depends on the characteristics of the individual oscillators, and on the nature and topology of the couplings that describe the interactions between oscillators. Perhaps the simplest representation of this phenomenon is the Kuramoto model^2, 3^, where individual units are described as pure phase-oscillators interacting through phase-dependent couplings and characterized by a natural frequency^4, 5^. In recent years, substantial emphasis has been put on the effects of the structure of the interaction network on synchronization^6–8^. Indeed, the interplay between the dynamical and structural properties of complex networks of oscillators can generate interesting phenomena, including explosive synchronization^9–11^ and the emergence of cluster synchronization^12, 13^.

Recent findings by Bergner *et al*.^14^ revealed an unexpected behavior dubbed “remote synchronization” (RS) in star-like networks of oscillators, whereby unconnected peripheral oscillators can synchronize through a hub that maintains free, independent dynamics. The occurrence of RS is intriguing and somewhat counterintuitive, as it implies synchronization of oscillators that are not directly connected by structural links, nor by chains of entrained oscillators. Hence, RS seems to entail a “hidden” transfer of information between remote nodes in the network through a hub that remains asynchronous. It should be noted that the term remote synchronization was also used in a different context by Nicosia *et al*. in ref. 15 where they describe a regime in which the oscillators in the whole network are in a stationary state and all have the same frequency (including hubs). Nicosia *et al*. refer to remote synchronization as a constant phase difference between otherwise synchronous oscillators. In the rest of this paper, with “remote synchronization” (RS) we refer to Bergner’s definition.

Remote synchronization has been demonstrated experimentally in simple models, like star^14^ or ring^16^ networks of oscillating electronic circuits, for example. Whether this phenomenon also plays a relevant role in natural networks, often characterized by the presence of hubs, or highly connected nodes in otherwise sparsely connected networks, remains the subject of active investigation^17–19^.

Here, we apply a model of coupled phase-oscillators to investigate the origin of remote synchronization. By introducing repulsive interactions between peripheral nodes, we demonstrate that the hub plays an active role in remote synchronization, rather than merely transferring information between peripheral nodes. Conversely, a hub can remotely desynchronize oscillators in the presence of a synchronizing mean field. These findings have important implications, as they show that the hub can actively influence the dynamics of the network’s nodes while remaining asynchronous itself.

We dub this phenomenon hub-driven remote synchronization and explore its role in networks endowed with hubs, a pervasive feature in many real-world networks. Using the Karate club network, a prototypical complex network^20^, we show that a degree-dependent distribution of natural frequencies results in complex patterns of remote synchronization, and that a shift in a single hub’s frequency can induce dramatic changes in synchronization patterns.

Finally, we study the conditions for this phenomenon to emerge in brain connectivity networks. Specifically, we leverage recent electrophysiological^21^ and structural connectivity data^22, 23^ to model the dynamics of spontaneous activity in the macaque brain, and demonstrate a potential role for hub-driven remote synchronization in shaping patterns of coherent activity, sometimes referred to as functional connectivity.

## Results

### Remote synchronization in a star network of Kuramoto oscillators

We adopt a Kuramoto-Sakaguchi model^24^

1 $${\dot{\phi }}_{i}={\omega }_{i}+\frac{\varepsilon }{{k}_{i}}\,\sum _{j=0}^{N}{a}_{ij}\,\sin ({\phi }_{j}-{\phi }_{i}-{\delta }_{ij}),\quad i=0\ldots N,$$

where ${k}_{i}={\sum }_{j\mathrm{=0}}^{N}{a}_{ij}$ is degree of the node *i*.

**Figure 1:**
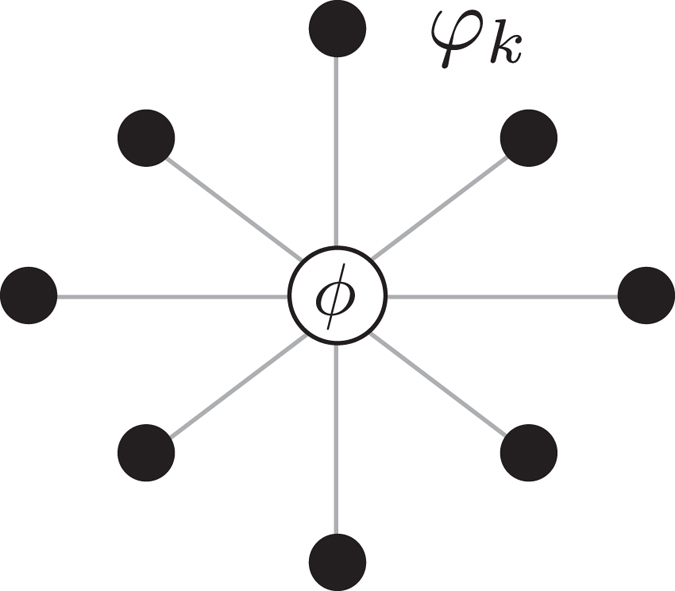
Configuration of a star network, where *ϕ* is a hub and *φ*_*k*_ are leaf oscillators, *k* = 1…*N*.

In case of star-like network (Fig. 1) we set *ϕ* = *φ*_0_ to be a hub and denote $A=\varepsilon {a}_{i0}$ and $\alpha ={\delta }_{i0}$ for *i* = 1… *N*, $B=\varepsilon {a}_{0j}$ and $\beta ={\delta }_{0j}$ for *j* = 1 … *N*. Thus, for identical phase oscillators described in ref. 25 the system (1) reads

2 $$\begin{array}{rcl}{\dot{\phi }}_{k} & = & \omega +A\,\sin \,(\varphi -{\phi }_{k}-\alpha ),\quad k=1\ldots N,\\ \dot{\varphi } & = & {\omega }_{0}+\frac{1}{N}\sum _{j\mathrm{=1}}^{N}B\,\sin ({\phi }_{j}-\beta -\varphi \mathrm{).}\end{array}$$

where *ϕ* denotes the phase of the hub (or leader) and *φ*_*k*_ the phases of the leaf oscillators. In this case, a synchronous solution can include constant phase differences between oscillators.

System (2) was analytically analyzed in ref. 25 by virtue of the Watanabe-Strogatz ansatz^26, 27^, a variable transformation akin to the Mobius transform (see section Methods for details). This method provides a description of the dynamics of a system like (2) through a complex global variable, namely the order parameter^28^. As shown in ref. 25, the dynamics of this system includes hysteretic transitions between asynchronous and synchronous states. Depending on model’s parameters, system (2) can have three different stable solutions.

(i). In the region of relatively small absolute values of frequency mismatch $|\omega -{\omega }_{0}|$, the system (2) has one stable synchronous solution that is stationary in the rotating reference frame. For this solution, the phases ${\phi }_{k}={\rm{\Phi }}$, $k=1\ldots N$ are identical, while the phase of the leader $\varphi ={\rm{\Phi }}-{\rm{\Delta }}{\rm{\Phi }}$, where ${\rm{\Delta }}{\rm{\Phi }}=\,{\rm{const}}$. Hence, the synchronous solution has non-zero but constant phase shift between the leader and the leaves.
(ii). With increasing frequency mismatch, an asynchronous regime emerges. Stability of the asynchronous solution depends on the sign of the expression ${\rm{sign}}(\sin (\alpha +\beta ))(\omega -{\omega }_{0})$. For positive values, the asynchronous solution is stable, while for negative values it becomes unstable.
(iii). When the frequency mismatch is too large to lock the phases of the leader and the leaves, the synchronous solution (i) turns into the RS regime, whereby all the leaf oscillators have same frequency and phase, while the leader’s frequency and phase are different. In ref. 25 this is called “synchronous limit cycle” solution. We note that this regime corresponds to the definition of RS according to Bergner *et al*.^14^. A specific condition for the RS regime to be stable is:

3 $${\rm{sign}}\,(\sin \,(\alpha +\beta ))(\omega -{\omega }_{0}) < -\sqrt{{A}^{2}+{B}^{2}+2AB\,\cos \,(\alpha +\beta )\,}\mathrm{.}$$

**Figure 2:**
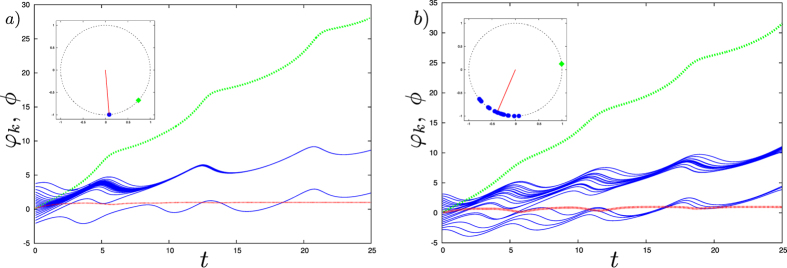
Simulation of the system (2) for *N* = 20 oscillators with the parameters *A* = *B* = 1, $\alpha =\beta =0.3\pi$ and ${\omega }_{0}=1.4$. In panel a) the natural frequencies of the oscillators are identical and set to 0; in panel b) *ω*_*i*_ are randomly chosen from the Gaussian distribution with the zero mean and the standard deviation 0.1. Time-dependence of the phase of the leader $\varphi (t)$ (dashed green line), the phases ${\phi }_{k}(t)$ of the leaves (solid blue lines) and their order parameter (dotted red line). Note that the difference between the blue branches is exactly 2*π*. (Insets) Snapshots of the oscillators. The leaves are represented as blue circles and the leader as a green diamond; the red line is the order parameter of the leaves.

Figure 2a demonstrates the RS solution of system (2) for the model parameters satisfying expression (3). This figure shows that, under these conditions, the difference between the synchronized leaves and the hub’s phases builds up in time. The Watanabe-Strogatz approach is applicable only if the natural frequencies of the peripheral nodes are identical; however the RS regime can be also observed in the inhomogeneous case, as illustrated on Fig. 2b.

From the brief description reported above and expression (3) it follows that the case of zero overall phase shift ($\alpha =-\beta$) is special. Indeed, stability analysis^25^ shows that in this case the asynchronous fixed point is neutrally stable. This implies that the RS regime is neutrally stable as well, thus making it difficult, if not impossible, to detect it numerically in a pure Kuramoto model with zero phase shift in the coupling term. This is the reason why the RS regime was not observed with pure Kuramoto oscillators in ref. 14 and 18, where the authors concluded that RS can only be observed in the presence of an additional degree of freedom, like amplitude in the Stuart-Landau equations. This additional degree of freedom was thought to be necessary for the appearance of RS regime, as it enables a hidden transfer of information through the amplitudes of the oscillators. However, the appearance of RS regime can arise from direct action of the hub on the leaves, as discussed below.

In summary, in this section we have presented the main results of ref. 25 and conclude that the regime therein described for Kuramoto oscillators corresponds to remote synchronization as defined by Bergner *et al*.^14^, in sharp contrast with their conclusion that remote synchrony cannot occur in pure phase oscillators’ systems^14, 18^.

### Hubs actively drive synchronization

In order to show that a leader (or hub) can have a direct synchronizing or desynchronizing effect on peripheral nodes depending on the model’s parameters, we consider a star network where the leaf oscillators are additionally subjected to a Kuramoto-Sakaguchi mean field. This system was introduced but not analyzed in ref. 25 in the form:

4 $$\begin{array}{rcl}{\dot{\phi }}_{k} & = & \omega +A\,\sin (\varphi -{\phi }_{k}-\alpha )+\frac{1}{N}\sum _{j\mathrm{=1}}^{N}C\,\sin ({\phi }_{j}-{\phi }_{k}-\gamma ),\quad k=1\ldots N,\\ \dot{\varphi } & = & {\omega }_{0}+\frac{1}{N}\sum _{j\mathrm{=1}}^{N}B\,\sin ({\phi }_{j}-\beta -\varphi \mathrm{).}\end{array}$$

System (4) can be obtained from (1) by setting $C=\varepsilon {a}_{ij}$ and $\gamma ={\delta }_{ij}$ for $i,j=1\ldots N$.

**Figure 3:**
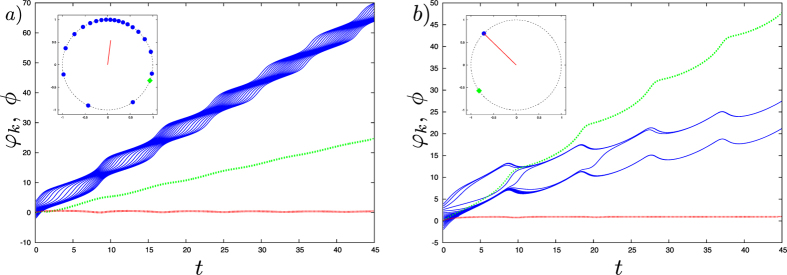
Simulation of system (4) for *N* = 20 oscillators with the parameters *A* = *B* = 1, $\alpha =\beta =0.3\pi$. In panel a) we show that an asynchronous hub can actively desynchronize the peripheral oscillators when an attractive mean-field is applied (*C* = 0.5, *γ* = 0.2*π*, *ω* = 2 and *ω*_0_ = 0.6). In panel b) we show the emergence of remote synchrony in the presence of a repulsive mean-field applied to the peripheral oscillators (*C* = 1, $\gamma =0.6\pi$, *ω* = 1 and *ω*_0_ = 1.4). Time-dependence of the phase of the leader $\varphi (t)$ (dashed green line), the phases ${\phi }_{k}(t)$ of the leaves (solid blue lines) and their order parameter (dotted red line). Note that the difference between the blue branches on panel b) is exactly 2*π*. (Insets) Snapshots of the oscillators. The leaves are represented as blue circles and the leader as a green diamond; the red line is the order parameter of the leaves.

Below we demonstrate that hub nodes can induce non-trivial regimes in the presence of attractive or repulsive mean fields. Specifically, we find:

(i) asynchronous solutions in the presence of an attractive ($\cos \,\gamma  > 0$) mean field. Figure 3a shows that in this regime the leader desynchronizes leaf oscillators in the bounded manifold of the positive frequency mismatch $\omega -{\omega }_{0}$.
(ii) Synchronous solutions in the presence of a repulsive ($\cos \,\gamma  < 0$) mean field. Figure 3b shows that the leader synchronizes the leaves in the bounded region of the negative frequency mismatch $\omega -{\omega }_{0}$. As in the conventional RS regime, the leader maintains free dynamics, asynchronous with respect to the leaves.

The above examples show that the leader can have a synchronizing action even in the presence of a desynchronizing mean field or, vice versa, a desynchronizing one in the presence of a synchronizing mean field. This is inconsistent with the notion that the hub serves the sole purpose of enabling information transfer between the peripheral nodes, and suggests that it directly drives the dynamics of the leaves.

By virtue of the Watanabe-Strogatz transformation, we performed a stability analysis of the synchronous solutions (see section Methods for details). The synchronous solution is stable if ${\lambda }_{s} < 0$ (5), with:

5 $${\lambda }_{s}=\{\begin{array}{cc}\frac{1}{u}(A\frac{B\,\sin \,\delta }{u}[\frac{\omega -{\omega }_{0}-C\,\sin \,\gamma }{u}+\sqrt{{(\frac{\omega -{\omega }_{0}-C\sin \gamma }{u})}^{2}-1\,}]-C\,\cos \,\gamma ), & \frac{\omega -{\omega }_{0}-C\,\sin \,\gamma }{u} < -1,\\ \frac{1}{u}(A[\frac{\omega -{\omega }_{0}-C\,\sin \,\gamma }{u}\cdot \frac{B\,\sin \,\delta }{u}-\sqrt{1-{(\frac{\omega -{\omega }_{0}-C\sin \gamma }{u})}^{2}\,}\frac{A+B\,\cos \,\delta }{u}]-C\,\cos \,\gamma ), & -1\le \frac{\omega -{\omega }_{0}-C\,\sin \,\gamma }{u}\le 1,\\ \frac{1}{u}(A\frac{B\,\sin \,\delta }{u}[\frac{\omega -{\omega }_{0}-C\,\sin \,\gamma }{u}-\sqrt{{(\frac{\omega -{\omega }_{0}-C\sin \gamma }{u})}^{2}-1\,}]-C\,\cos \,\gamma ), & \frac{\omega -{\omega }_{0}-C\,\sin \,\gamma }{u} > 1,\end{array}$$

where $$u=\sqrt{{A}^{2}+{B}^{2}+2AB\,\cos \,\delta }$$.

**Figure 4:**
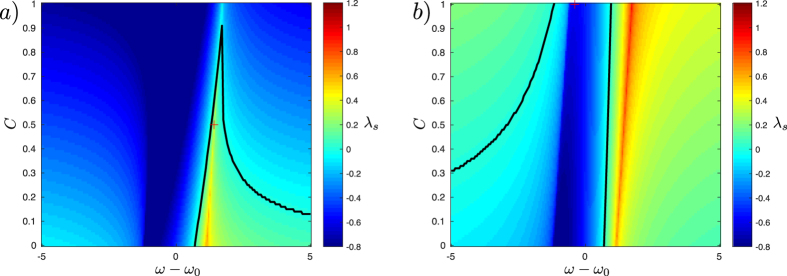
*λ*_*s*_ (as in Eq. (5)) as a function of the frequency mismatch *ω*–*ω*_0_ and the strength *C* of the mean field for the case *A* = *B* = 1, *α* = *β* = 0.3*π*. The synchronous solution is stable if *λ*_*s*_ < 0. The black line represents the stability border (*λ*_*s*_ = 0) for *γ* = 0.2*π* (**a**) and *γ* = 0.6*π* (**b**). The red crosses in (**a**) and (**b**) show the parameters used in Fig. 3a and b, respectively.

From expression (5) it follows that even for relatively large mean-field strength the hub counteracts the effects of the field when its frequency mismatch falls in a certain range (Fig. 4). For attractive mean-fields, active hub-driven desynchronization is observed for positive frequency mismatches ($\omega -{\omega }_{0} > 0$, with *ω*_0_ indicating the natural frequency of the hub). Vice versa, negative mismatches can drive synchronization in the presence of repulsive mean-field.

### Hub-driven remote synchronization in a prototypical complex network

In order to investigate hub-driven remote synchrony in more complex systems, we implement the Kuramoto-Sakaguchi model (1) on the Karate club network^20^, a widely studied social network. This network represents a middle ground between a star-like toy model and more complex real world networks, and possesses clearly identifiable hub nodes.

As shown in the previous section, if the sine of the cumulative phase shift is positive, the frequencies of the hubs must be sufficiently larger than the frequencies of the peripheral oscillators (leaves) to observe the RS regime. By way of example, we adopt the distribution of frequencies suggested in ref. 9, where the frequency of the oscillator is proportional to the degree of the node ${\omega }_{i}={k}_{i}$ (by rescaling time the coefficient of proportionality can be absorbed in the coupling strength *ε*). This distribution of frequencies has no physical meaning for this particular network, and was taken solely to demonstrate the possibility of a RS regime in a small complex network with hub nodes.

As a measure of synchrony between nodes, we take the time-averaged order parameter called synchronization index:

6 $${r}_{ij}=|{\langle {e}^{{\rm{i}}[{\phi }_{i}(t)-{\phi }_{j}(t)]}\rangle }_{t}|,$$

where ${\langle \cdot \rangle }_{t}$ denotes an average over large period of time. Note that this order parameter is not sensitive to constant phase shifts, and takes large values when the average frequencies of the two oscillators are similar. We consider nodes *i* and *j* to be synchronized if ${r}_{ij} > 0.75$, a value that corresponds to a minimum in the histogram of node-wise synchronization indices. Other thresholds in the range 0.7–0.9 produce qualitatively and quantitatively similar results.

**Figure 5:**
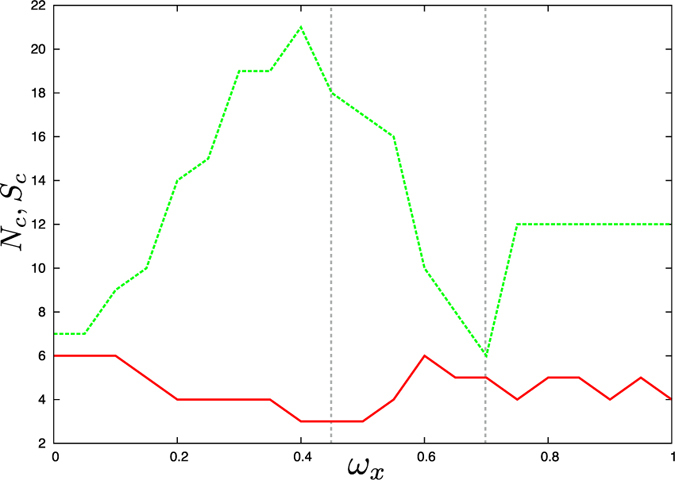
Dependence of the number of synchronized clusters *N*_*c*_ (solid red line) and size of the largest cluster *S*_*c*_ (dashed green line) on the hub frequency multiplier *ω*_*x*_. The parameters are *ε* = 5, *δ* = 0.2*π*. For increasing values of the frequency multiplier, the size of the largest synchronized component presents a bell-shaped behavior, with the largest synchronized cluster observed for *ω*_*x*_ = 0.4.

In order to study how the frequencies of the hubs affect synchronization of the network a multiplier *ω*_*x*_ is introduced to selectively vary the frequencies of the two hub nodes (33 and 34). Figure 5 shows the number of synchronized clusters of nodes *N*_*c*_ and the size of the largest cluster *S*_*c*_ as a function of *ω*_*x*_.

For each value of *ω*_*x*_, several numerical simulations were performed with different, random initial conditions. With increasing *ω*_*x*_ the size of the largest cluster follows a bell shaped curve, with a maximum corresponding to *ω*_*x*_ = 0.4. Conversely, the number of clusters shows a complementary behavior, albeit less pronounced.

**Figure 6:**
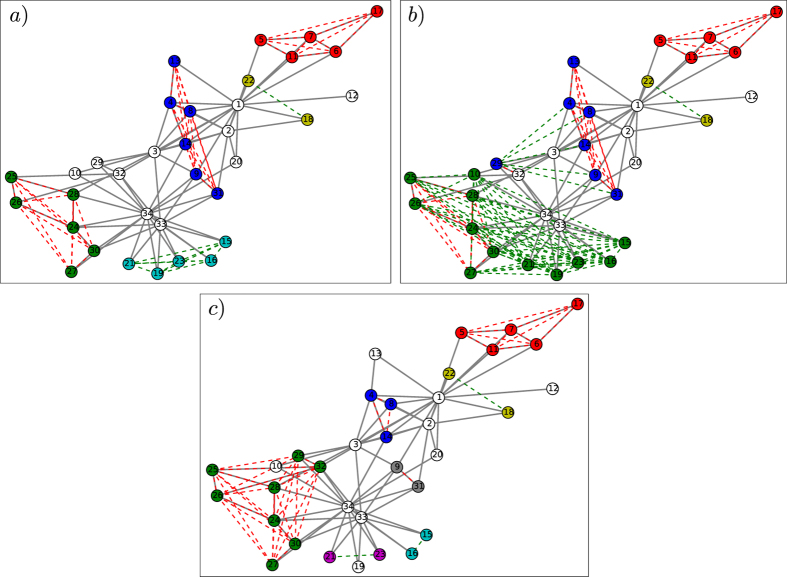
Synchronization patterns in the Karate club network for different values of parameters. The edges of the Karate club network are shown by solid grey connections. Remote and direct synchrony links with ${r}_{ij}\mathrm{ > 0.75}$ are shown by dashed green and red lines, respectively. Clusters of synchronized nodes are indicated by different colors of the full circles. For *ω*_*x*_ = 0.7, *δ* = 0.2*π* clusters of remote synchrony start to emerge (panel a). For larger values of the hubs’ natural frequencies *ω*_*x*_ = 1, *δ* = 0.2*π*, a large cluster of remotely synchronized nodes appears, as the leaf oscillators of hubs 33 and 34 become synchronized (panel b). Remote synchrony is hardly observed when *δ* = 0, *ω*_*x*_ = 1 (panel c).

Patterns of synchronization on the Karate-club network for different values of *ω*_*x*_ are shown in Fig. 6, where grey lines denote structural links, and red and green dashed lines connect nodes that are directly or remotely synchronized, respectively. In the case of non-zero phase shifts *δ*, we note that the highly connected hubs 33 and 34 generate a cluster of remotely synchronized nodes while remaining asynchronous.

At *ω*_*x*_ = 0.7 remote synchronization starts to emerge, comprising small clusters of nodes (Fig. 6a). Above *ω*_*x*_ = 0.7, the frequencies of the hubs are large enough to remotely synchronize their leaf nodes and the size of the remotely synchronized cluster increases sharply (Fig. 6b). We observed a similar behavior also for other hub nodes, like node 1 (data not shown), thus suggesting that the mechanism is not idiosyncratic to the particular configuration of node 33 and 34, but is generally applicable to hubs.

In the case of zero phase shift *δ* = 0, occasional pairwise correlations may appear (e.g. between nodes 15 and 16 in Fig. 6c) depending on the initial conditions, but extended clusters of remotely synchronized oscillators do not emerge.

In summary, hub-driven remote synchrony is not limited to toy star-like networks, but can be found in more complex networks endowed with hubs, like the Karate club network. In this model, we found that synchronization patterns strongly depend on the hubs’ natural frequencies.

### Hub-driven remote synchronization in brain connectivity networks

The interplay between structural connections and synchronization of dynamical processes on networks is reminiscent of the concept of functional connectivity in the realm of neuroscience. In this context, functional connectivity is defined in terms of correlations or coherence between oscillatory behaviors observed, e.g., in the electrical or hemodynamic spontaneous activity of the brain.

The question we intend to address in the following is whether the conditions for remote synchronization exist in brain networks and may play a role in determining patterns of correlated activity as observed, e.g., in EEG or MEG neuroimaging experiments in the brain under resting conditions. The key ingredients for this phenomenon to emerge are: (i) the presence of hubs within brain networks of structural connectivity; (ii) a phase shift between remote nodes resulting from a delay in the interaction terms; (iii) a difference in the oscillatory frequency of hubs with respect to their peripheral nodes.

A number of studies (see refs 29 and 30 for recent reviews) based on Diffusion Tensor Magnetic Resonance Imaging have shown that certain brain regions are characterized by high degree and “betweenness”, i.e. they act as gateways of many shortest paths connecting pairs of nodes. These connector hubs play an important role in the integration of the network and in ensuring efficient transfer of information across the graph. This has been observed in humans^31^ as well as in other species, including non-human primates and rodents^32^. *Ex-vivo* studies using anterograde or retrograde tracers corroborate this evidence in experimental laboratory animals like the macaque. Hence, condition (i) appears to be fulfilled.

Finite signal propagation speed in axons generates distance-dependent time delays in the interaction terms in brain networks^33–35^. In a model of coupled phase oscillators, these delays can be represented as phase shifts proportional to internodal distance^33, 36^, as in condition (ii).

Finally, condition (iii) has been recently addressed in a meta-analysis of electrophysiology experiments in the macaque, showing anatomical dependence of spontaneous oscillations of populations of neurons in a number of brain areas as measured by invasive electrophysiology^21^. Comparison with degree distribution in macaque^32^ shows that the fast nodes from^21^ are also structural hubs.

In the light of this evidence, we have modeled the synchronization phenomenon in the macaque brain using experimental data and empirically determined parameters from the literature.

For our simulations, we adopted the structural connectivity graph described in^22, 23^, where connectivity data was obtained by retrograde tracer injections in 29 areas of the macaque cerebral cortex. Extrinsic fraction of labeled neurons (the ratio between the number of labeled neurons in the source area over the total number of labeled cortical neurons extrinsic to the injected area) for each pathway determines the weight of the connection between areas. The 29 by 29 connectivity matrix was thresholded by percolation analysis of the giant component^37–40^ and binarized^41^.

Simulations were performed based on this connectivity graph by adopting the model (1) without normalization of the coupling strengths by node degree. Following^25, 33, 42^ we assumed that the phase shifts in the coupling terms are proportional to the distances between nodes. Internodal distances were taken from ref. 23 as the length of the shortest trajectory interconnecting areas via the white matter, approximating the axonal distance.

Murray *et al*.^21^ collected measurements of timescales of intrinsic fluctuations in spiking activity in different areas of the macaque brain. In ref. 43, the selection of regions was extended using modeling of the macaque neocortex. Specifically, Chaudhuri *et al*.^43^ calculated autocorrelation functions of activity in response to white-noise input to all areas. Time constants of the decay of autocorrelation were calculated for all nodes included in our model. The dominant time constants in various areas of the network were extracted by fitting single or double exponentials to the autocorrelation. In case of double exponentials, the timescales of the two components were calculated as the weighted average of the two time constants. For the frequencies in our model we took inverse timescales.

**Figure 7:**
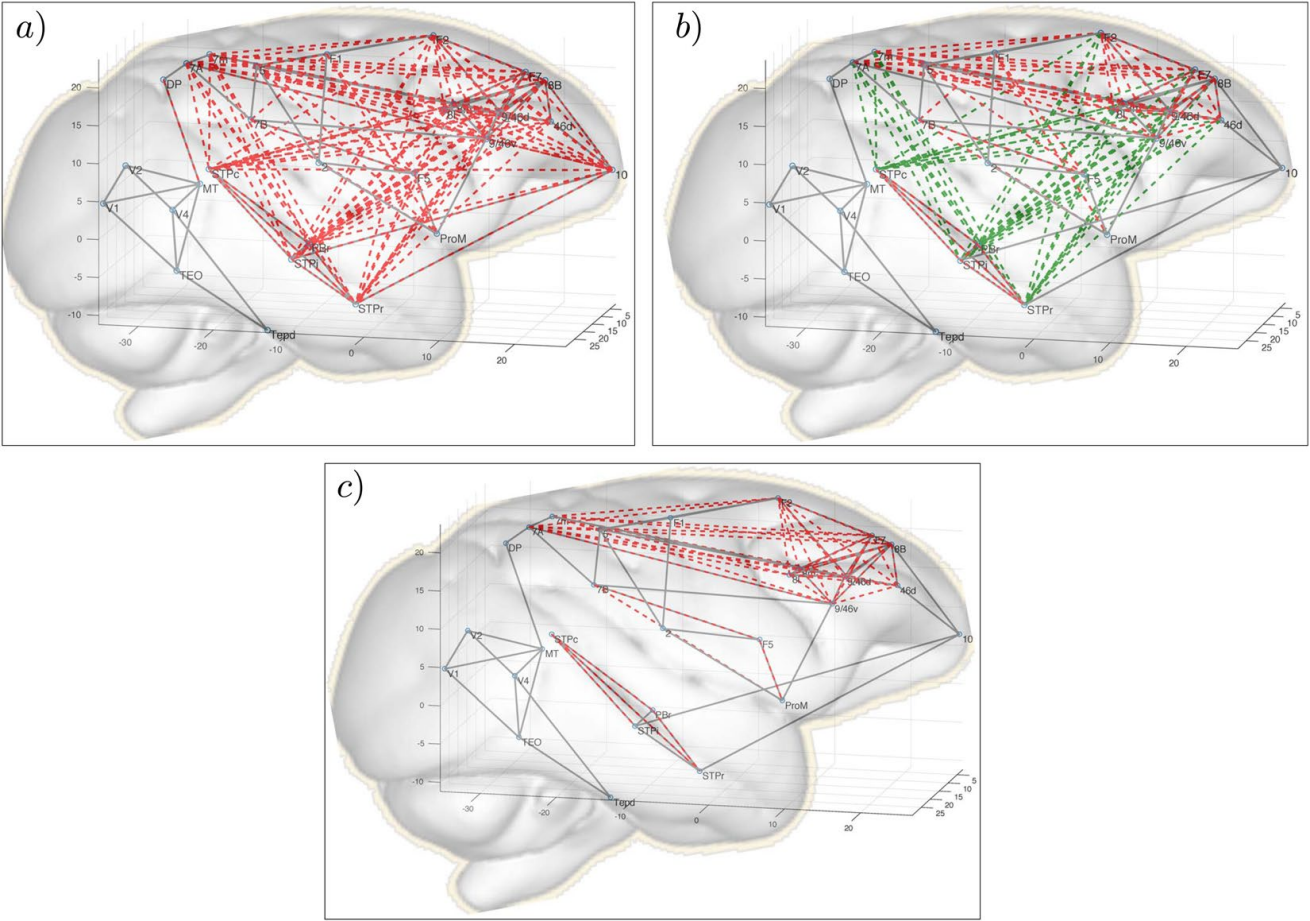
Simulation of the Macaque connectome. Structural links are shown with grey lines. Remote and direct synchrony links with ${r}_{ij} > 0.75$ are shown by dashed green and red lines, respectively. The parameters are $\varepsilon =0.85$, ${\delta }_{ij}=2\pi {D}_{ij}{\nu }_{s}/c$, where ${\nu }_{s}=40$ Hz, *c* = 10 m/s and *D*_*ij*_ is the internodal distance matrix taken from ref. 23. Frequencies are taken as inverse timescales for resting-state case from ref. 43. For ${\omega }_{10}=\mathrm{1/3.17}$ (panel a), node 10 is synchronous with a large cluster of nodes in the frontal and temporal cortices. When we increase the natural frequency of node 10 to its fast component (${\omega }_{10}=\mathrm{1/0.185}$), the hub becomes asynchronous while driving remote synchronization of fronto-temporal regions (panel b). Increasing the frequency further (${\omega }_{10}=\mathrm{1/0.0625}$) frontal and temporal regions become functionally disconnected (panel c).

The results of our simulations are shown on Fig. 7. In the structural connectivity network, a node in the prefrontal cortex denoted as 10 plays the role of structural hub that connects areas in the frontal and temporal cortices. Using the frequency distribution calculated from ref. 43, we obtain a wide pattern of synchronization (red dashed lines), including all frontal and temporal cortices (Fig. 7a). When we switch the frequency of node 10 to that of the fast component associated with this node^43^, coherent synchronization among those areas is preserved, despite the fact that node 10 becomes asynchronous with the rest of the cluster, consistent with the definition of remote synchronization (green dashed lines in Fig. 7b). When we further increase the frequency of the oscillator associated with node 10, frontal and temporal areas become functionally disconnected, and form two separate clusters of synchronized nodes. Hence, a switch between the regime of normal synchronization (Fig. 7a), remote synchronization (Fig. 7b) and asynchrony (Fig. 7c) can be driven by a single parameter in the model, namely the hub’s frequency.

This finding is particularly interesting as it may indicate a new and potentially important mechanism in the emergence of functional connectivity patterns in the brain. Here, we have shown that the appearance of clusters of synchronized areas can be driven by structural hub regions that do not appear to be functionally connected to these areas. This is consistent with the observation that structural and functional hubs in the brain do not necessarily coincide^44, 45^. Importantly, changes in the frequency of spontaneous fluctuations in a hub region can dynamically reconfigure patterns of functional connectivity in the brain, switching between the regimes of direct and remote synchronization, or actively desynchronizing functional modules even when couplings between nodes are attractive. Changes in spontaneous fluctuations may occur in response to external stimuli, or perhaps changes in brain state, resulting in a reconfiguration of the synchronization pattern.

While the factors enabling hub-driven RS appear to be present in the brain, a role of this phenomenon in shaping functional connectivity patterns remains to be proven. However, this hypothesis can be experimentally tested. By way of example, it may be envisaged that optogenetic technology^46^, whereby photosensitive proteins are expressed in specific populations of neurons, could provide a means to drive the oscillatory behavior of selected hub regions while measuring patterns of synchronized activity across the brain.

## Discussion and Conclusion

In the first part of this paper we have summarized the analytical results obtained in ref. 25 for the Kuramoto-Sakaguchi model on star networks. Specifically, we have explicitly shown that “remote synchronization” (RS), in Bergner’s definition^14^, can be observed in systems of Kuramoto phase oscillators if a non-zero phase shift is introduced in the coupling terms. For pure star networks, the RS regime is stable when a sufficiently large frequency mismatch is imposed between leaves and the hub. Importantly, we have shown that the hub can exert a synchronizing action even in the presence of additional repulsive mean field acting on the peripheral nodes. Hence, the hub does not simply enable transfer of information between the leaves, but actively drives synchronization while remaining asynchronous with the rest of the network. Conversely, the hub can actively desynchronize the leaves in the presence of an attractive mean field. We have analytically derived the conditions whereby these phenomena can occur in a star network by a stability analysis of the synchronous solutions.

In the second part we have explored the role of this mechanism in the synchronization of an exemplary complex network. As an example, we have chosen the Karate-club network, a prototypical social network in which a few highly connected individuals play the role of hubs. When a degree-dependent distribution of natural frequencies is introduced in the model, remote synchrony emerges and plays a substantial role in the formation of clusters of synchronized nodes within the network.

Finally, we have explored the potential role of this mechanism in brain connectivity networks. Indeed, the prerequisites for hub-driven remote synchronization appear to exist in the brain, including the presence of structural hubs, delays in the couplings between nodes, and region-dependent frequency of spontaneous fluctuations. We have leveraged connectomic data from the macaque brain and recent electrophysiological measurements to derive a dynamical model of synchronization in the macaque cortex. Our simulations show that a change in the intrinsic frequency of a hub can dramatically reshape synchronization patterns, shifting from direct to remote synchronization, and to a hub-driven desynchronization regime. This experimentally testable hypothesis may explain the mismatch between structural and functional hubs sometimes observed in brain connectivity networks.

## Methods

### The Watanabe-Strogatz approach

The Watanabe-Strogatz (WS) theory^26, 27^ can be applied to any system of the form

7 $${\dot{\phi }}_{k}=f(t)+{\rm{I}}{\rm{m}}(F(t){e}^{-{\rm{i}}{\phi }_{k}}),$$

where *f*(*t*) is an arbitrary real and *F*(*t*) is an arbitrary complex functions. Note that a global coupling can enter one or both functions.

The dynamics of the general system (7) can be characterized by one global complex variable $z=z(t)$ and one real global variable ${\rm{\Psi }}={\rm{\Psi }}(t)$ and *N* constants of motion ${\psi }_{k}$ (of which only *N* − 3 are independent) by virtue of WS variable transformation

8 $${e}^{{\rm{i}}{\phi }_{k}}=\frac{z+{e}^{{\rm{i}}({\psi }_{k}+{\rm{\Psi }})}}{1+{z}^{\ast }{e}^{{\rm{i}}({\psi }_{k}+{\rm{\Psi }})}},$$

with additional constraints ${\sum }_{i}{e}^{{\rm{i}}{\psi }_{i}}={\sum }_{i}\,\cos \,2{\psi }_{i}=0$. Equations for *z*(*t*) and Ψ(*t*) are obtained by substituting (8) into the system (7).

9 $$\begin{array}{rcl}\dot{z} & = & {\rm{i}}f(t)z+\frac{F(t)}{2}-\frac{{F}^{\ast }(t)}{2}{z}^{2},\\ \dot{{\rm{\Psi }}} & = & f(t)+\text{Im}({z}^{\ast }F(t\mathrm{)).}\end{array}$$

Where *z*(0) and ${\rm{\Psi }}\mathrm{(0)}$ together with the constants ${\psi }_{k}$ are determined by initial conditions of original variables ${\phi }_{k}\mathrm{(0)}$.

### Stability analysis

Stability analysis of the system (4) is performed with the help of WS theory presented above. Following^25^ we perform variable transformation to the phase differences ${\rm{\Delta }}{\phi }_{k}$

10 $${\rm{\Delta }}{\phi }_{k}={\phi }_{k}-\varphi +\alpha \mathrm{.}$$

The system for ${\rm{\Delta }}{\phi }_{k}$ reads

11 $$\frac{d{\rm{\Delta }}{\phi }_{k}}{dt}=\omega -{\omega }_{0}-{\rm{I}}{\rm{m}}(G(t))+{\rm{I}}{\rm{m}}(A{e}^{-i{\rm{\Delta }}{\phi }_{k}})+{\rm{I}}{\rm{m}}(H(t){e}^{-i{\rm{\Delta }}{\phi }_{k}}),$$

where global fields are introduced in the following way:

12 $$\begin{array}{rcl}G(t) & = & B{e}^{-{\rm{i}}(\alpha +\beta )}\frac{1}{N}\sum _{j\mathrm{=1}}^{N}{e}^{i{\rm{\Delta }}{\phi }_{j}},\\ H(t) & = & C{e}^{-{\rm{i}}\gamma }\frac{1}{N}\sum _{j\mathrm{=1}}^{N}{e}^{i{\rm{\Delta }}{\phi }_{j}}\mathrm{.}\end{array}$$

Direct comparing of the systems (11) and (7) gives $f(t)=\omega -{\omega }_{0}-\text{Im}(G(t))$ and $F(t)=A+H(t)$.

As shown in refs 25 and 28, in the thermodynamic limit $N\to \infty$ and for a uniform distribution of constants of motion *ψ* the order parameter $Z(t)=\frac{1}{N}{\sum }_{j=1}^{N}{e}^{i{\rm{\Delta }}{\phi }_{j}}$ is equal to $z(t)$. In ref. 47 it was demonstrated that, in case of small perturbations, initially non-uniform distributions of constants tend toward the vicinity of the uniform one. Therefore, in this case

13 $$\begin{array}{rcl}G(t) & = & B{e}^{-{\rm{i}}(\alpha +\beta )}z(t),\\ H(t) & = & C{e}^{-{\rm{i}}\gamma }z(t\mathrm{).}\end{array}$$

From the expressions (13) for the global fields, it is apparent that Ψ(*t*) does not enter the equation for *z*(*t*) in (9). Thus the equation for *z*(*t*) (14) describes the dynamics of the system (11) and consequently of the original system (4).

14 $$\dot{z}={\rm{i}}(\omega -{\omega }_{0}-B\,\text{Im}(z{e}^{-{\rm{i}}\delta }))z-A\frac{{z}^{2}-1}{2}+\frac{C}{2}({e}^{-{\rm{i}}\gamma }-{e}^{{\rm{i}}\gamma }|z{|}^{2})z.$$

As in ref. 25 it is convenient to perform rescaling of time (15)

15 $$t^{\prime} =t\sqrt{{A}^{2}+{B}^{2}+2AB\,\cos \,\delta \,}\mathrm{.}$$

and reparametrization (16)

16 $${\rm{\Delta }}x=\frac{\omega -{\omega }_{0}}{\sqrt{{A}^{2}+{B}^{2}+2AB\,\cos \,\delta \,}\,}\,{\rm{and}}\,\sin \,\xi =\frac{A+B\,\cos \,\delta }{\sqrt{{A}^{2}+{B}^{2}+2AB\,\cos \,\delta }}\mathrm{.}$$

Then the equations for the magnitude *ρ* and the argument ΔΦ of the order parameter $z=\rho {e}^{i{\rm{\Delta }}{\rm{\Phi }}}$ are

17 $$\begin{array}{rcl}\frac{d\rho }{dt} & = & \frac{1-{\rho }^{2}}{2}(g\,\cos \,{\rm{\Delta }}{\rm{\Phi }}+q\,\cos \,\gamma \,\rho ),\\ \frac{d{\rm{\Delta }}{\rm{\Phi }}}{dt} & = & {\rm{\Delta }}x-q\frac{1+{\rho }^{2}}{2}\,\sin \,\gamma +\,\cos \,\xi \rho \,\cos \,{\rm{\Delta }}{\rm{\Phi }}-\frac{g+\mathrm{(2}\,\sin \,\xi -g){\rho }^{2}}{2\rho }\,\sin \,{\rm{\Delta }}{\rm{\Phi }},\end{array}$$

where $$g=\frac{A}{\sqrt{{A}^{2}+{B}^{2}+2AB\,\cos \,\delta }}\ge 0$$ and $$q=\frac{C}{\sqrt{{A}^{2}+{B}^{2}+2AB\,\cos \,\delta }}\ge 0$$

For the synchronous solution $\rho =|z|=1$

18 $$\begin{array}{rcl}\frac{d\rho }{dt} & = & \mathrm{0,}\\ \frac{d{\rm{\Delta }}{\rm{\Phi }}}{dt} & = & {\rm{\Delta }}x-q\,\sin \,\gamma +cos\,\xi \,\cos \,{\rm{\Delta }}{\rm{\Phi }}-\,\sin \,\xi \,\sin \,{\rm{\Delta }}{\rm{\Phi }},\end{array}$$

If $|{\rm{\Delta }}x-q\,\sin \,\gamma |\le 1$ the steady solutions for ΔΦ are

19 $${{\rm{\Delta }}{\rm{\Phi }}}_{s1}=\frac{\pi }{2}+{\rm{arc}}\,\sin ({\rm{\Delta }}x-q\,\sin \,\gamma )-\xi ,\,{{\rm{\Delta }}{\rm{\Phi }}}_{s2}=-\frac{\pi }{2}-{\rm{arc}}\,\sin ({\rm{\Delta }}x-q\,\sin \,\gamma )-\xi \mathrm{.}$$

These steady solutions (19) are stable if

20 $${\lambda }_{s1,2}=g[({\rm{\Delta }}x-q\,\sin \,\gamma )\cos \,\xi \mp \sqrt{1-{({\rm{\Delta }}x-q\sin \gamma )}^{2}\,}\sin \xi ]-q\,\cos \,\gamma  < 0,$$

where − corresponds to ${{\rm{\Delta }}{\rm{\Phi }}}_{s1}$ and + to ${{\rm{\Delta }}{\rm{\Phi }}}_{s2}$.

If $|{\rm{\Delta }}x-q\,sin\,\gamma | > 1$ the regime analogous to remote synchronization can be observed, which is stable if

21 $${\lambda }_{s}=g\,\cos \,\xi [({\rm{\Delta }}x-q\,\sin \,\gamma )\mp \sqrt{{({\rm{\Delta }}x-q\sin \gamma )}^{2}-1}]-q\,\cos \,\gamma  < 0,$$

where − corresponds to ${\rm{\Delta }}x-q\,\sin \,\gamma  > 1$ and $+$ to $\Delta x-qsin\gamma  < -1$.

Taking into account that out of the two steady solutions only ΔΦ_*s*1_ is of interest and returning to the original parameters, we obtain the expression (5) for *λ*_*s*_.

### Experimental data

Experimental connectivity data for the macaque brain was taken from ref. 22, internodal distances were taken from ref. 23. The list of the abbreviation of the brain regions (from ref. 22): 2 - somatosensory area 2; 5 - somatosensory area 5; 7A - area 7A; 7B - area 7B; 7m - area 7m; 8B - area 8B; 8l - lateral part of area 8; 8m - medial part of area 8; 9/46d - area 9/46, dorsal part; 9/46v - area 9/46, ventral part; 10 - area 10; 24c - area 24c; 46d - area 46, dorsal part; DP - dorsal prelunate area; F1 - frontal area F1; F2 - frontal area F2; F5 - frontal area F5; F7 - frontal area F7; MT - middle temporal area; PBr - parabelt, rostral part; ProM - area ProM; STPc - superior temporal polysensory, caudal part; STPi - superior temporal polysensory, intermediate part; STPr - superior temporal polysensory, rostral part; TEO - area TEO; TEpd - area TE, posterior-dorsal part; V1 - visual area 1; V2 - visual area 2; V4 - visual area 4.
